# Dispositional mindfulness mediates the relationships of parental attachment to posttraumatic stress disorder and academic burnout in adolescents following the Yancheng tornado

**DOI:** 10.1080/20008198.2018.1472989

**Published:** 2018-05-17

**Authors:** Yuanyuan An, Guangzhe Yuan, Zhen Liu, Yuyang Zhou, Wei Xu

**Affiliations:** a School of Psychology, Nanjing Normal University, Nanjing, P. R. China; b Shanghai Mental Health Center, Shanghai Jiao Tong University School of Medicine, Shanghai, P. R. China; c Shanghai International Studies University, Shanghai, P. R. China

**Keywords:** Parental attachment, dispositional mindfulness, PTSD, academic burnout, • We found that parental attachment and dispositional mindfulness are both negatively correlated with PTSD and academic burnout.• We found that parental attachment and dispositional mindfulness are both negatively correlated with academic burnout.•We found that dispositional mindfulness mediates the relationships between parental attachment and PTSD and academic burnout.

## Abstract

**Background**: Previous studies have shown that parental attachment is associated with low severity of posttraumatic stress disorder (PTSD) and low academic burnout in individuals who have experienced traumatic events.

**Objective**: The present study investigated the ways in which parental attachment is related to PTSD symptoms and academic burnout in Chinese traumatized adolescents by considering the role of dispositional mindfulness.

**Method**: A total of 443 Chinese adolescents who had experienced a severe tornado one year prior to this study completed measures of parental attachment, dispositional mindfulness, PTSD and academic burnout.

**Results**: The results showed that our model fitted the data well [*χ^2^/df* = 2.968, CFI = 0.971, TLI = 0.955, RMSEA (90% CI) = 0.067 (0.052–0.082)] and revealed that dispositional mindfulness partially mediates the relationship between parental attachment, PTSD severity and academic burnout.

**Conclusions**: The findings suggested that dispositional mindfulness and parental attachment may be two critical resources in dealing with traumatization and academic burnout.

## El mindfulness disposicional media las relaciones del apego parental con Trastorno de Estrés Postraumático y burnout académico en adolescentes después del tornado de Yancheng

**Antecedentes**: Estudios previos han demostrado que el apego parental se asocia con una baja gravedad del trastorno de estrés postraumático (TEPT) y bajo burnout académico en individuos que han experimentado eventos traumáticos.

**Objetivo**: El presente estudio investigó las formas en que el apego parental se relaciona con los síntomas de TEPT y el burnout académico en adolescentes chinos traumatizados al considerar el rol del mindfulness disposicional.

**Método**: Un total de 443 adolescentes chinos que habían experimentado un tornado severo 1 año antes de este estudio completaron medidas de apego parental, mindfulness disposicional, trastorno de estrés postraumático y burnout académico.

**Resultados**: Los resultados mostraron que nuestro modelo se ajustaba bien a los datos [χ^2^/df = 2.968, CFI = 0.971, TLI = 0.955, RMSEA (90% IC) = 0.067 (0.052–0.082)] y reveló que el mindfulness disposicional media parcialmente la relación entre el apego parental, la gravedad del TEPT y el burnout académico.

**Conclusiones**: Los hallazgos sugirieron que el mindfulness disposicional y el apego parental pueden ser dos recursos críticos para lidiar con la traumatización y el burnout académico.

## 盐城龙卷风后, 特质正念调节父母依恋与创伤后应激障碍和青少年学业倦怠的关系

**背景**： 以前的研究表明，父母依恋与创伤后事件个体的创伤后应激障碍（PTSD）较低严重程度和较低的学业倦怠相关。

**目的**： 本研究考察了父母依恋与遭受创伤的中国青少年创伤后应激障碍症状和学业倦怠的关系，并考虑了特质倾向的作用。

**方法**： 在本研究前1年经历过严重龙卷风的443名中国青少年中，测量了父母依恋、特质正念、创伤后应激障碍和学业倦怠。

**结果**： 结果表明，我们的模型很好地拟合了数据[c2/df = 2.968，CFI = 0.971，TLI = 0.955，RMSEA（90％CI）= 0.067（0.052–0.082）]，并且显示特质正念部分地调节了父母依恋，PTSD严重程度和学业倦怠之间的关系。

**结论**： 发现表明，特质正念和父母依恋可能是应对创伤和学业倦怠的两个关键资源。

## 1. Introduction

A severe tornado occurred in Yancheng city, Jiangsu province, China, in the summer of 2016. The disaster progressed rapidly within a day and left the city destroyed. It is estimated that the tornado killed 99 people, injured approximately 800 and more than 1.6 million were affected (Lyu, Wang, Cheng, & Shen, 2017). Beyond the physical, environmental and economic toll, catastrophic disasters cause mental health problems to individuals who experience a disaster directly or who live in the affected community (Houston et al., 2015). In a review of 160 disaster studies, Norris and her colleagues (2002) concluded that youth were at higher risk than adults from mental health problems following a traumatic event. Therefore, adolescents are generally in greater need of help and support.

### 1.1. Posttraumatic stress disorder

Posttraumatic stress disorder (PTSD) is one of the most studied negative outcomes in trauma-focused research (Fan, Long, Zhou, Zheng, & Liu, 2015; Giannopoulou et al., 2006). Youth are at particularly high risk of being exposed to traumatic events (Lieberman & Van Horn, 2009). In a systematic review of natural disaster studies, Neria, Nandi, and Galea (2008) found that the prevalence of PTSD among direct victims of disasters ranges is 30–40%. One study sampled adolescents who were exposed to the Wenchuan earthquake. Rates for high-risk PTSD were 24.3% in a mild exposure group and 38.6% in a severe exposure group (Wang et al., 2012). Other researchers have reported that the rates of posttraumatic stress symptoms (PTSS) among the Wenchuan earthquake survivors ranged from 27.7 to 79.3% (Xu & Song, 2011). Many researchers argued that traumatized adolescents, when unattended, may develop PTSD and psychological symptoms that carry into adulthood, such as depression, anxiety and substance abuse (Fan, Zhang, Yang, Mo, & Liu, 2011; Kilpatrick et al., 2003). Empirical evidence that advances our understanding of PTSD in traumatized adolescents is crucial for preventive intervention programmes in this research area.

### 1.2. Academic burnout

As with PTSD symptoms, considerable attention has been paid to adolescents’ academic burnout following trauma (Xu et al., 2017; Ying, Wang, Lin, & Chen, 2016). Academic burnout can be characterized by emotional exhaustion in study demands, possession of detached attitudes toward academic tasks and displays of reduced efficiency or academic competence (Maslach, Schaufeli, & Leiter, 2001). It may be particularly prevalent among adolescents exposed to natural disasters because experiencing stressful life events (such as natural disasters) is one important risk factor of academic burnout (Lin & Huang, 2014; Mather, Blom, & Svedberg, 2014). In Lin et al.’s (2013) research, 828 primary and secondary school students reported academic burnout, especially impairment in learning self-efficiency, 30 months after the 2008 Wenchuan earthquake. Similarly, Xu and his colleagues (2017) found that many students reported varying degrees of academic burnout within the area of the Yancheng tornado. Adolescents experiencing major life events are prone to have academic burnout (Dyrbye et al., 2006; Ying et al., 2016), thus academic burnout can also be found among traumatized students (Xu et al., 2017). However, the experience of disasters does not affect everyone in the same way (Birkeland, Hansen, Blix, Solberg, & Heir, 2017).

Adolescents not only show PTSD but also report academic burnout following traumatic events. Many researchers have suggested that PTSD and burnout may be coexistent in people following stressful environments (Mealer, Burnham, Goode, Rothbaum, & Moss, 2009; Zhou, Zhen, & Wu, 2017). Hence, it is important to explore the potential protective factors of PTSD and academic burnout following exposure to natural disasters. In the current study, we examined the role of parental attachment as one potential protective factor in a sample of adolescents in Yancheng following the 2016 Yancheng tornado.

### 1.3. Parental attachment, PTSD and academic burnout

There is no doubt that traumatic experience is a precondition for posttraumatic reactions, but not all traumatized adolescents report PTSD symptoms (Zhou, Wu, Fu, & An, 2015) or academic burnout (Ying et al., 2016). Therefore, it is hypothesized that other factors must affect the occurrence of posttraumatic reactions (Freedy, Resnick, & Kilpatrick, 1992). According to attachment theory, the quality of attachment relationships determines the individual’s internal working models of self and others (Bowlby, 1969). These working models vary on a continuum of security vs. insecurity and influence emotion regulation and how individuals cope with stressors (Bartholomew & Horowitz, 1991; Sroufe & Waters, 1977). Nolen-Hoeksema and Watkin’s (2011) heuristic, for developing transdiagnostic models of psychopathology of identifying the mechanisms linking distal risk factors (e.g. early parental environment) to proximal risk factors (e.g. emotional, cognitive or behavioural tendencies) and proximal risk factors to psychopathology, shows that distal risk factors contribute to disorders by mediating proximal risk factors and refers to the possible causal process linking attachment orientation to disorders. Thus, one candidate variable that is likely to be associated with PTSD symptoms and academic burnout is parental attachment (Aspelmeier, Elliott, & Smith, 2007; Duchesne & Larose, 2007; Han, 2006). Lack of stress-reducing secure attachment often results in PTSD in adolescents (Benoit, Bouthillier, Moss, Rousseau, & Brunet, 2010; Dieperink, Leskela, Thuras, & Engdahl, 2001). Many researchers have theorized and investigated parental attachment as a resiliency factor for traumatized children and adolescents (e.g. Cicchetti & Carlson, 1989; Lieberman, Weston, & Pawl, 1991). Among a sample of 1153 Chinese adolescents 8.5 years after Wenchuan earthquake, Tian, Wu, Wang, and Zhou (2018) found that secure parental attachment negatively predicted PTSD. A growing body of literature has documented that there is a close relation between attachment patterns and PTSD symptoms (Arikan, Stopa, Carnelley, & Karl, 2016). The causal role of attachment-related process in the development of PTSD was examined in research with a prospective design (Mikulincer, Shaver, & Horesh, 2006). By using three Israelis samples who had experienced terrorist attacks, some researchers found that attachment anxiety was a unique predictor of posttraumatic stress and trauma-related psychological problems (Besser & Neria, 2012, 2010; Besser, Neria, & Haynes, 2009). To our knowledge, few researchers investigated the relationship between attachment and PTSD among traumatized individuals. Moreover, using a sample of 121 French adolescents, Duchesne and Larose (2007) found that adolescents’ attachment to both parents was positively associated with academic motivation, whilst academic motivation can be a negative predictive factor of academic burnout (Yao, 2009). Since we found that there are close relations between parental attachment, PTSD and academic burnout, this study aimed to examine the predictive mechanisms by considering the mediating role of dispositional mindfulness.

### 1.4. Parental attachment and mindfulness

Mindfulness refers to one’s general tendency to be able to attend to the present moment nonjudgmentally and purposefully (Brown & Ryan, 2003). As one’s state of mindfulness may be unstable and sensitive to change, dispositional mindfulness has been frequently assessed in the literature (Chiesa, Calati, & Serretti, 2011; Garland, Gaylord, & Fredrickson, 2011). In the field of attachment theory, through longitudinal studies covering decades of development, researchers have demonstrated that internal attunement of a parent to a child enables the child’s mind to become resilient (Bowlby, 2003; Cassidy & Shaver, 2010). Securely attached children can balance their emotions well, meet their intellectual potential, develop their capacity of having meaningful relationships with others and thus improve their development of dispositional mindfulness (Argus & Thompson, 2008; Hülsheger, Alberts, Feinholdt, & Lang, 2013). Based on the models of Goodall, Trejnowska, and Darling (2012) and Pepping, Davis, and O’Donovan (2013), secure attachment could be a protective factor for dispositional mindfulness. Ryan, Brown, and Creswell (2007) argued that attachment security may provide an individual with a greater capacity to maintain mindful attention and awareness, as they would be less consumed by factors related to insecure attachment such as rumination or avoidance. Moreover, studies in secure attachment and mindfulness have a remarkable convergence in outcome measures (Kabat-Zinn, 2003; Sroufe, Egeland, Carlson, & Collins, 2005) and both are associated with functions of the middle aspects of the prefrontal cortex (Siegel, 2007). While extensive research investigating the direct relationship between attachments and dispositional mindfulness among adolescents is not yet available, a preliminary study by DiNoble (2009) has found that adults with secure attachments have increased scores on mindfulness traits.

### 1.5. Dispositional mindfulness, PTSD and academic burnout

Considerable attention has been paid to mindfulness following trauma (e.g. Chopko & Schwartz, 2009; Follette, Palm, & Pearson, 2006; Thompson, Arnkoff, & Glass, 2011). A vast body of literature from interventions and associational studies has demonstrated the health benefits of mindfulness in both clinical and community samples (Brown & Ryan, 2003; Grossman, Niemann, Schmidt, & Walach, 2004; Khusid & Vythilingam, 2016). Based on a mindfulness stress-buffering hypothesis, which posits that mindfulness mitigates stress appraisals and reduces stress reactivity responses, and that these stress reduction effects partly or entirely explain how mindfulness affects mental and physical health outcomes, mindfulness could influence posttraumatic stress symptoms (Creswell, 2014, pp. 426–427). Some preliminary evidence has pointed to the negative relationship between dispositional mindfulness and PTSD symptoms. For example, self-reported dispositional mindfulness has been found to be inversely associated with PTSD symptoms in college students (Thompson et al., 2011). In addition, many researchers found that dispositional mindfulness was a predictive factor of mental health problems (Boden et al., 2012; Boughner, Thornley, Kharlas, & Frewen, 2016; Hagen, Lien, Hauff, & Heir, 2016; Smith et al., 2011; Thompson & Waltz, 2010).

Even though the protective role of mindfulness in reducing burnout and posttraumatic symptoms has been revealed (separately) in past research (Cohen-Katz, Wiley, Capuano, Baker, & Shapiro, 2005; Flook, Goldberg, Pinger, Bonus, & Davidson, 2013; Thompson & Waltz, 2010), few studies have examined the relationship between dispositional mindfulness and academic burnout among traumatized adolescents. Xu et al. (2017), the only study concerning the association between dispositional mindfulness and academic burnout, found that dispositional mindfulness can negatively predict academic burnout in a sample of 247 Chinese adolescents. Similar results were found in several other types of burnout. Using a sample of 64 educators, Abenavoli, Jennings, Greenberg, Harris, and Katz (2013) showed that educators’ mindfulness had strong, consistent negative associations with job burnout. In Gustafsson, Skoog, Davis, Kenttä, and Haberl (2015), bivariate correlations revealed that mindfulness had a significant negative relationship with burnout among 233 adolescent athletes. Thus, dispositional mindfulness may predict PTSD symptoms and academic burnout in a sample of Chinese adolescent survivors following severe trauma.

### 1.6. The current study

We propose that parental attachment can relieve PTSD symptoms and academic burnout by considering the mediating role of dispositional mindfulness among traumatized adolescents following a severe disaster. This role was examined in the current study using a sample of 443 Chinese adolescents. Participants were junior high school students in Yancheng, China, who had experienced a significant tornado one year before. Putting these rationales together, two hypotheses were generated: (a) parental attachment has direct negative associations with PTSD and academic burnout and (b) dispositional mindfulness would mediate the relationship between parental attachment and both PTSD and academic burnout.

## 2. Method

### 2.1. Participants and procedures

The participants in the study were adolescents from two junior high school in Yancheng, Jiangsu, China, where the tornado took place. A total of 455 adolescents were recruited. Excluding the invalid answers from 12 participants, data from 443 participants were available for analysis. The mean age of the participants was 14.44 (*SD* = 0.72) and 208 (47.0%) participants were male. All participants experienced this traumatic event; nine of the participants were trapped when the tornado came and six were injured. Seventy-eight participants’ relatives or friends were trapped and 80 participants’ relatives or friends were injured. Twenty-seven participants’ relatives or friends died.

This study was approved by the Ethic Committee of the School of Psychology, Nanjing Normal University. In the current study, written informed consent was obtained from school principals and classroom teachers.^1^ The assessment was conducted under the supervision of the researchers. The questionnaires were completed class by class, and participant classes were randomly selected in each school. Students completed the pencil-and-paper questionnaires in quiet classrooms and it took them approximately 20 minutes to finish all questionnaires. During the testing session, no student was absent. After that, the researchers conducted a 10-minute group games session as the reward for their involvement. After the questionnaires were completed, we provided them with contact information for reliable psychological consultancy organizations and a list of self-help psychology books. They were encouraged to seek help if they need mental health assistance.

### 2.2. Measures

#### 2.2.1. PTSD

PTSD symptom levels were measured by the Child PTSD Symptom Scale (CPSS; Foa, Johnson, Feeny, & Treadwell, 2001) which evaluates Diagnostic and Statistical Manual of Mental Disorders-IV (DSM-IV) PTSD symptoms on occurrence and frequency. This scale has 17 items rated on a 4-point Likert scale from 0 (not at all/only once) to 3 (almost always). This scale was applied to the Chinese population with good validity and reliability (Ying, Wu, & Chen, 2013; Ying, Wu, Lin, & Jiang, 2014). In the current study, all items were translated into Chinese. Participants rated their current experiences of PTSD symptoms during the previous two weeks. The subscale scores range from 0–15 for intrusion symptoms (e.g. ‘I have nightmares’), 0–21 for avoidance symptoms (e.g. ‘I lost interests in many things that I enjoyed doing’) and 0–15 for hyperarousal symptoms (e.g. ‘I have trouble falling or staying asleep’). An overall severity score was generated by summing the scores of the three symptom types. Using confirmatory factor analysis (CFA), Zang (2010) found an adequate fit of a model with the three subscales [*χ* ^2^ (116, *N* = 225) = 234.89], root mean-square error of approximation (RMSEA = .070, comparative fit index = .095, normed-fit index = .094). In this sample, the scale demonstrated good internal consistency (α = .89).

#### 2.2.2. Academic burnout

The Learning Burnout Questionnaire (LBQ) is a 21-item scale developed in a Chinese student sample to assess academic burnout (Hu & Dai, 2007). Items were rated on a 5-point scale ranging from 0 (not at all) to 4 (almost always). The LBQ includes four subscales: mental exhaustion, the lack of personal learning accomplishment, the alienated relationship between students and teachers and physical exhaustion. Examples of the items included ‘I don’t care whether I finished my homework’, ‘I have poor sleep’ and ‘I don’t want to study’. Higher scores indicated greater severity of academic burnout. In this sample, the scale demonstrated good internal consistency (α = .83).

#### 2.2.3. Parental attachment

The revised version of Inventory of Parent and Peer Attachment (IPPA-R) was used to measure participants’ attachment to parents. This scale consists of 25 items rated on a 5-point scale (from 1 = ‘never’ to 5 = ‘always’) assessing the magnitude of trust, communication and alienation towards parents. Higher scores on trust and communication and lower score on alienation indicate better parental attachment. Examples of the items included ‘I am angry at my parents’ and ‘My parents respect my feelings’. All items were reverse scored to compute a composite score such that higher scores indicate higher levels of parental attachment. This scale has shown good reliability and construct validity in several Chinese adolescent samples (Li, Delvecchio, Lis, Nie, & Di Riso, 2015; Song, Thompson, & Ferrer, 2009). In this sample, the scale demonstrated good internal consistency (α = .88).

#### 2.2.4. Dispositional mindfulness

Dispositional mindfulness was measured by the Chinese version of the Mindfulness Attention Awareness Scale (MAAS; Deng et al., 2012). The original MAAS (Brown & Ryan, 2003) is a widely-used scale to assess an individual’s dispositional mindfulness. It has 15 items, each rated on a 6-point scale from 1 (always) to 6 (never). Examples of the items included ‘I find myself doing things without paying attention’, ‘I find it difficult to stay focused on what’s happening in the present’ and ‘I rush through activities without being really attentive to them’. All items were reverse scored to compute a composite score such that higher scores indicate higher levels of dispositional mindfulness. This scale has shown good reliability and construct validity in an adolescent sample (Black et al., 2012). In this sample, the scale demonstrated good internal consistency (α = .93).

### 2.3. Data analysis

The analyses were conducted using SPSS 22.0 and AMOS 21.0. We found that 1.57% of data was missing. Little’s Missing Completely at Random (MCAR) test suggested that the rate of missing data was equivalent across all measures (*p* > .05). Descriptive statistics were used to calculate the mean levels of the main measures and Pearson’s correlations were used to assess the relations between parental attachment, mindfulness, PTSD and academic burnout. Structural equation modelling was performed using maximum likelihood estimation. In the mediation model, parental attachment is the independent variable. Dispositional mindfulness is the proposed mediator. PTSD and academic burnout are both outcome variables. To test the hypothesized mediation effect for statistical significance by AMOS, boot strapping was applied using 5000 bootstrap samples. The indices that measured the model’s goodness-of-fit included the ratio of *χ* ^2^ to the degree of freedom, goodness-of-fit index (GFI), comparative fix index (CFI) and root mean square error of approximation (RMSEA). For an index to be acceptable, the ratio of *χ* ^2^ to the degree of freedom should not exceed 3, GFI should exceed .9, CFI should exceed .95 and RMSEA should be smaller than .08 (Hu & Bentler, 1999).

## 3. Results

We first applied Harman’s single-factor test to examine common method bias (Podsakoff, MacKenzie, Lee, & Podsakoff, 2003). All items relevant to the study were subjected to an exploratory factor analysis, and the un-rotated factor solution was examined to determine the number of factors that are necessary to account for the overall variance. This procedure suggested 11 factors, while no single factor accounted for the majority of the covariance among the variables. Therefore, no significant common method bias existed in the current study.

Means, standard deviations and Cronbach’s alpha coefficients of the measures are shown in Table 1. All measures have good or acceptable reliability. Table 1 shows significant correlations between parental attachment, dispositional mindfulness, PTSD symptoms and academic burnout.

**Table 1.** Means, standard deviations (*SD*), Cronbach’s alpha coefficients and correlations of all the variables (*N* = 443).

Variables	*M* ± *SD*	α	1	2	3	4
1. Parental attachment	80.69 ± 16.44	.88	-	.28***	−.28***	−.50***
2. Dispositional mindfulness	64.45 ± 16.28	.93	.28***	-	−.52***	−.45***
3. PTSD	11.15 ± 8.11	.89	−.28***	−.52***	-	−.46***
4. Academic burnout	28.46 ± 14.32	.83	−.50***	−.46***	.46***	-

Note: The numbers in lower left are the correlation coefficient without controlling age and gender, the numbers in top right are the correlation coefficient after controlling age and gender. PTSD = posttraumatic stress disorder.

***p < .001.

We built a measurement model that included three latent variable constructs: parental attachment, PTSD symptoms and academic burnout. The parental attachment latent variable was evaluated through the scores for three subscales of trust, communication and alienation. The PTSD latent variable was evaluated by the scores of three subscales, namely intrusion, avoidance and hyper-arousal. Additionally, the academic burnout latent variable was evaluated by the scores of four subscales: mental exhaustion, the lack of personal learning accomplishment, the alienated relationship between students and teachers and physical exhaustion. In this measurement model, correlations were specified among parental attachment, PTSD and academic burnout. Factor loadings for the manifest indicators on their respective latent variables were estimated freely. The model was found to fit the data well [*χ^2^/df* = 3.447, CFI = 0.969, TLI = 0.950, RMSEA (90% CI) = 0.074 (0.058–0.091)]. The measurement model was sound and suitable for further analysis of the structural equation model.

Based on this measurement model, we then built a direct effect model that demonstrated the effects of parental attachment on PTSD and academic burnout. This model also fit the data well [*χ^2^/df* = 3.487, CFI = 0.968, TLI = 0.949, RMSEA (90% CI) = 0.075 (0.059–0.091)]. Path analyses revealed that parental attachment was a significant negative predictor of PTSD and academic burnout.

**Figure 1.**
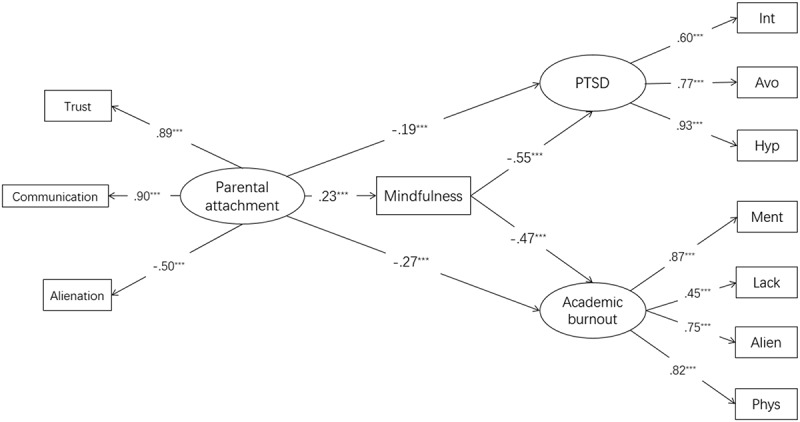
Mediation model (*N* = 443) with standardized beta weights and significance levels added. Note: PTSD = posttraumatic stress disorder; Int = Intrusion; Avo = Avoidance; Hyp = Hyper arousal; Ment = Mental exhaustion; Lack = the lack of personal learning accomplishment; Alien = the alienated relationship; Phys = Physical exhaustion. ***p < .001.

Based on the direct effect model, we inserted mindfulness between parental attachment and PTSD, as well as between parental attachment and academic burnout to establish an indirect effect model (see Figure 1). We found that this indirect effect model also fit the data well [*χ^2^/df* = 2.968, CFI = 0.971, TLI = 0.955, RMSEA (90% CI) = 0.067 (0.052–0.082)]. Figure 1 shows the path coefficients of the model. All path coefficients were statistically significant. More importantly, bootstrap analyses showed that the relationship between parental attachment and PTSD symptoms was mediated by dispositional mindfulness (with the indirect effect = − 0.126, 95% CI = − 0.188 ~ −0.077), and the relationship between parental attachment and academic burnout was also mediated by dispositional mindfulness (with the indirect effect = − 0.107, 95% CI = − 0.156 ~ −0.065). The standardized direct and indirect effects are reported in Table 2 (with 95% CI using the bootstrap method).

**Table 2.** Standardized direct, indirect and total effects of variables PTSD symptoms and academic burnout.

	*Β*	95% CI	*p*	*β*	95% CI	*p*	*β*	95% CI	*p*
**PTSD symptoms**									
Parental attachment	−.188	−.278/-.102	***	−.126	−.188/-.077	***	−.315	−.420/-.212	***
Mindfulness	−.547	−.635/-.455	***	0	–	–	−.547	−.635/-.455	***
**Academic burnout**									
Parental attachment	−.271	−.372/-.171	***	−.107	−.156/-.065	***	−.379	−.483/-.269	***
Mindfulness	−.465	−.554/-.367	***	0	–	–	−.465	−.554/-.367	***
**Mindfulness**									
Parental attachment	.231	.138/.326	***	0	–	–	.231	.138/.326	***

Note. CI = confidential interval; PTSD = posttraumatic stress disorder.

***p < .001.

## 4. Discussion

To our knowledge, this is the first study to examine the roles of parental attachment and dispositional mindfulness simultaneously in predicting PTSD symptoms and academic burnout. This study found that parental attachment had indirect negative associations with PTSD symptoms and academic burnout mediated by dispositional mindfulness.

Specifically, and consistent with mindfulness stress-buffering hypothesis and previous research (Besser & Neria, 2012, 2010; Besser et al., 2009; Duchesne & Larose, 2007), our direct effect model results suggest that parental attachment negatively predicts PTSD and academic burnout. These results also indicate that parental attachment can play a positive role in mental health and academic motivation among adolescents, regardless of stressful experience had by an individual.

Furthermore, consistent with previous research (Waters, Merrick, Treboux, Crowell, & Albersheim, 2000), the parental attachment was positively related to dispositional mindfulness among adolescents. The indirect effect model indicates that the direct effect of parental attachment on PTSD and academic burnout is still significant when mindfulness was inserted into the relation between parental attachment and PTSD and academic. Based on the Nolen-Hoeksema and Watkins (2011) heuristic for developing transdiagnostic models of psychopathology, low parental attachment, as a distal risk factor, would contribute to PTSD symptoms and academic burnout through the mediating role of mindfulness (a proximal risk factor). Thus, compared with traumatized adolescents with lower levels of parental attachment, those with higher levels of parental attachment were more likely to have better dispositional mindfulness, which could be helpful to alleviate their PTSD symptoms and academic burnout. This study provides early evidence that traumatized adolescents with high levels of parental attachment engage in less PTSD symptoms and academic burnout through the development of mindfulness.

Several design and measurement limitations must be acknowledged. First, all variables were measured by self-report scales. Thus, the associations between the main measures might be affected by ‘systematic error variance shared among variables measured with and introduced as a function of the same method and/or source’ (Richardson, Simmering, & Sturman, 2009, p. 763), and lead to some potential bias in estimating associations. Future studies should involve gathering data through multiple methods. Second, because this study was conducted one year after the Yancheng tornado, the participants may have experienced other traumatic events after the tornado. Third, this study overlooked that parental attachment and dispositional mindfulness might exist prior the tornado and acted as resiliency factors, and these factors might be affected by trauma exposure. Fourth, trauma severity and reflective function – which refers to the capacity to reflect on one’s own thoughts and feelings – might influence dispositional mindfulness and should be considered. Moreover, this study was a cross-sectional study and thus only non-causal attributions can be made.

Despite these limitations, this study contributes new knowledge to previous theoretical and empirical work on the relationships between parental attachment, dispositional mindfulness, PTSS symptoms and academic burnout. This study not only highlighted attachment to parents as a potentially crucial coping resource in the context of traumatization, but also integrated Nolen-Hoeksema and Watkins’ (2011) transdiagnostic model, extending the applicability of these theories to adolescent tornado survivors. In terms of intervention and health-enhancement, this study also highlights significant implications for adolescent survivors of the Yancheng tornado. Clinical efforts should focus on the protection and improvement of parental attachment. For example, parents can provide emotional and material support for adolescents and work to foster a supportive environment. Additionally, helping adolescents to raise the level of mindfulness and encouraging them to hold more positive attitudes towards traumatic events as well as posttraumatic emotions may mitigate PTSD symptoms and academic burnout after trauma.

## Note 1.

In China, when researchers recruit students as study participants where the research will be conducted in a school environment, ban zhu ren teachers (translated as ‘parentis teachers’) or school principals act as guardians for the students and supervise the research. Thus, a common practice is to use child or adolescent students’ consents without requiring their parents’ consents.
